# Colonic Anastomoses Reinforced With Ethyl-2-Cyanoacrylate Compared With Polydioxanone Sutures Alone in Fecal Peritonitis: An Experimental Study in Wistar Rats

**DOI:** 10.7759/cureus.49516

**Published:** 2023-11-27

**Authors:** David Ponce-Herrera, Sol Ramírez-Ochoa, Efrén Flores-Álvarez, Ramiro Gómez-Arambulo, José M Nava-Román, Alma G Méndez-Esparza, Gabino Cervantes-Guevara, Alejandro González-Ojeda, Clotilde Fuentes-Orozco, Carlos M González-Valencia, Mauricio A Ambriz-Alarcón, Héctor Meugniot-García, Brian R Rubio-Mora, Enrique Cervantes-Pérez

**Affiliations:** 1 Department of Surgical Oncology, Hospital Civil de Guadalajara Fray Antonio Alcalde, Guadalajara, MEX; 2 Department of Internal Medicine, Hospital Civil de Guadalajara Fray Antonio Alcalde, Guadalajara, MEX; 3 Department of Surgical Oncology, Centenario Hospital Miguel Hidalgo, Aguascalientes, MEX; 4 Department of Surgery, Centenario Hospital Miguel Hidalgo, Aguascalientes, MEX; 5 Department of Surgery, ISSEA Hospital General Tercer Milenio, Aguascalientes, MEX; 6 Department of Surgery, Hospital MAC Aguascalientes, Aguascalientes, MEX; 7 Department of Welfare and Sustainable Development, Centro Universitario del Norte, Universidad de Guadalajara, Guadalajara, MEX; 8 Department of Gastroenterology, Hospital Civil de Guadalajara Fray Antonio Alcalde, Guadalajara, MEX; 9 Biomedical Research Unit 02, Centro Médico Nacional de Occidente Instituto Mexicano del Seguro Social, Guadalajara, MEX; 10 Department of Research Ethics, Hospital Hispano de Especialidades, Guadalajara, MEX; 11 Department of Internal Medicine, Centro Médico Nacional de Occidente Instituto Mexicano del Seguro Social, Guadalajara, MEX; 12 Tlajomulco University Center, Universidad de Guadalajara, Tlajomulco de Zuñiga, MEX

**Keywords:** anastomoses reinforcement, abdominal sepsis, faecal peritonitis, colonic surgery, anastomotic dehiscence

## Abstract

Introduction: The use of tissue adhesives has been proposed as an anastomosis reinforcement; however, their efficacy has not been evaluated in a contaminated environment. The objective of this study was to determine if the use of sutures reinforced with ethyl-2-cyanoacrylate for colonic anastomoses in the presence of fecal peritonitis, in a murine animal model, decreases the frequency of dehiscence.

Methods: Wistar rats were used. Fecal peritonitis was established until reaching 18 hours of evolution. Then, resection and anastomosis of the colon were performed with only polydioxanone (PDS) sutures in the control group and reinforcement with ethyl-2-cyanoacrylate in the experimental group. The dehiscence frequency and burst pressure were evaluated six days after the anastomosis was performed.

Results: We included 30 Wistar rats, all males, with a median age of five months and an average weight of 350.43 g. Anastomotic dehiscence was observed in 53.33% of the control group, in contrast with 13.33% of the experimental group (p = 0.020). There was no significant difference in burst pressure between the two groups.

Conclusion: The use of ethyl-2-cyanoacrylate, in an experimental murine animal model, as reinforcement in colonic anastomoses in the presence of fecal peritonitis decreases the frequency of anastomotic dehiscence, although it does not increase resistance to burst pressure.

## Introduction

Currently, intestinal anastomoses have improved in terms of techniques and results to solve various problems, both in elective and emergency surgery [1], due to their low leak rates in colorectal surgery (between 3% and 23%) secondary to surgical technological advances, such as stapling techniques, intraoperative air tests, and direct vision sigmoidoscopy [2,3]. Many factors are involved and can contribute to the development of complications in colorectal surgery, both positively and negatively influencing the healing of the anastomosis. These factors can be general (advanced age, malnutrition, cancer), local (intraabdominal infection, low oxygen supply to the tissue, radiation), or systemic (chemotherapy, uremia, diabetes, steroid use) [1,3-5]. Among the complications, the main adverse event is dehiscence; its incidence varies from 10% to 50% using conventional sutures and is associated with high morbidity and mortality (50% and 20%, respectively) [1,5].

Currently, the performance of intestinal anastomoses in patients with fecal peritonitis, known as high risk, remains controversial; however, various strategies and modifications to conventional techniques have been developed for the prevention of complications [6-8], as despite a better understanding of the pathophysiology, advances in diagnosis, surgery, antimicrobial therapy, and management in the intensive care unit, peritonitis remains a potentially fatal condition [9]. Regarding the origin of the infectious source and the nature of the microbial contamination, peritonitis can be classified according to the Hamburg classification, and elsewhere, as primary, secondary, and tertiary [9-11].

Intestinal anastomoses have been successfully performed for more than 150 years using a wide variety of techniques, materials, and devices [12]. Several studies have demonstrated the safety of performing a primary anastomosis in the presence of peritonitis [13], but the time between the presentation of peritonitis and the anastomosis procedure variable of this condition continues to be a risk factor for anastomosis dehiscence, which is why controversy about management still exists [13]. A proof of concept study was carried out by Espinoza-Anguiano [14], in which it was shown that, after the induction of peritonitis in Wistar rats, the longer the time elapsed between the induction of peritonitis and the performance of the anastomosis, the greater the proportion of rats that presented dehiscence of the anastomosis in each group (16.6% of rats in the group where anastomosis was performed four to six hours after peritonitis induction presented dehiscence, in the group it was performed at 10 hours, 50% rats presented dehiscence, and in the groups it was performed at 16 and 24 hours, 66.6% rats presented dehiscence [14].

Suture-induced disruptions to the microcirculation can lead to transmural and mucosal necrosis causing anastomotic leaks [15,16]. Although epithelialization begins immediately after completion of the anastomosis, the tensile strength of the anastomosis is very low due to decreased collagen content and changes in collagen composition during postoperative days 2-7 [15,16]. The tensile strength of the anastomosis decreases to a nadir on postoperative day 4 and gradually increases thereafter. During this period of low resistance, the retention capacity of the suture determines the stability and integrity of the anastomosis. Therefore, sutures can be considered a technical dilemma that holds the anastomosis together but causes inflammation that may play a role in the occurrence of unwanted complications in the early postoperative period [16].

Currently, various suture substitutes have been used, such as staples and adhesive glues [17-19]. Despite these advances, there is still a high incidence of dehiscence in high-risk anastomoses [4,20], defined as those performed in an emergency situation, in developed or inflamed tissue, with the presence of ischemic patches, in unsafe anatomical conditions, or in malnourished patients with a reduced capacity for adequate healing [4,20].

A prospective study by Hyman et al. evaluated the outcome of 1223 patients who underwent resection and anastomosis without prior bypass [21]. The incidence of anastomotic leakage was 2.7% (33 cases) in that population [21]. The ideal suture material for an anastomosis is one that retains its resistance with complete integrity throughout the healing process; it should also cause minimal tissue reaction, avoid infection, and exhibit easy handling [16].

Cyanoacrylates (CAs) were first synthesized in 1949 [22], while their adhesive properties and possible use as surgical adhesives were first described in 1959 [19]. The first product developed after methyl-CA was ethyl-CA (C6H7NO2), which is soluble in methyl-ethyl-ketone and toluene and is already defined as a synthetic tissue adhesive (TA), that is, nonbiological, which, when in contact with a surface and through an exothermic reaction, sticks together, forming a highly resistant cover [18,22].

Since biological tissues contain many protein base residues, CAs are extremely adhesive to them, given their good moisture potential [18,19,22,23]. As an extension to this main property, CAs in biological applications also add the benefits of a hemostatic and bacteriostatic effect on gram-positive bacteria by forming an impermeable occlusive layer [18,19,22,23]. As a TA, CAs have three basic characteristics: (a) great adhesion resistance; (b) biocompatibility; and (c) biodegradability, in addition to great ease of application and stability [18,19]. Their fundamental disadvantage is the rapidity of their effect, which makes it practically impossible to rectify the surfaces to be joined [18,19].

Many studies have been carried out in search of the necessary tools to increase safety in the performance of intestinal anastomoses in high-risk patients [13]. One of the main situations for surgeon involvement is when a patient has peritonitis secondary to left colon disease, whether benign (complicated diverticular disease), malignant (colon cancer), or traumatic (closed or penetrating abdominal trauma). That said, there are studies that demonstrate the safety of performing a primary anastomosis in the presence of peritonitis [13].

Multiple studies have been performed with the use of TAs in intestinal anastomoses [15,20,24]. Their use as the sole method of anastomosis or as reinforcement for a suture line has been evaluated. The results shown in these studies are highly variable; some of them report a benefit in healing and increased burst pressure, while others report little or no benefit from the procedure [15,20,24]. Tebala et al. demonstrated the effectiveness of N-butyl-2-CA in reinforcing anastomoses made with staplers or sutures [20]. Elemen et al. showed no difference in intestinal anastomoses in rats but highlighted the advantage of a shorter operating time [15]. Faion et al. demonstrated the effectiveness of ethyl-2-CA as a sealant for the management of cecal stumps [24].

The efficacy of using TAs to reinforce intestinal anastomoses in patients with peritonitis of more than 12 hours of evolution has not been evaluated. If favorable in relation to the reduction of anastomotic dehiscence, the use of TAs would have a great impact on the patient because the condition could be resolved from the first intervention; moreover, the hospital stay, as well as the cost of health services, would decrease since reintervention in a second surgery to restore intestinal transit is avoided [21,25].

Measuring burst pressure is a method commonly used to assess the efficacy of various suture materials in animal models [26]. Burst pressure measurement involves the expansion of the intestine. When used to assess the mechanical strength of an anastomosis, the area where the segments are joined should be filled with gas or liquid until a leak occurs. The pressure recorded immediately before the occurrence of the leak is considered the burst pressure [26]. The magnitude of this pressure exhibits significant variability among different animal models and even within each individual experiment. The variation in sealing procedures, the condition of the animal before sacrifice, and the status of the colon in each experimental model contribute to this difference in pressure. Consequently, there is no universally accepted or typical range of measurements [26]. Therefore, the values are typically only relevant to the specific experimental model from which they were derived. The objective of conducting this measurement is to obtain a comparative assessment of the mechanical strength of the anastomosis. This allows for the evaluation of various suture or adhesive techniques and facilitates meaningful comparisons between them. It is important to note that the observed values cannot be directly translated into a clinical equivalent [26,27].

The objective of this study was to evaluate the efficacy of ethyl-2-CA as a reinforcement for sutures in comparison with sutures alone to reduce the dehiscence of anastomoses in the presence of fecal peritonitis in a mouse model.

## Materials and methods

This study was conducted at the Surgical Techniques Laboratory, Universidad Autónoma de Aguascalientes, in Aguascalientes city, Mexico. It was approved by the Internal Ethics Committee for the Care and Use of Laboratory Animals of the Autonomous University of Aguascalientes (approval number: CEI65/CI38/18) and carried out in accordance with the Official Mexican Standard NOM-062-ZOO-1999, and with international regulations and recommendations.

Thirty healthy, male, Wistar rats, four to six months old, weighing between 280 and 420 g, were used in the study. The animals were single-housed in Makrolon® (Covestro AG, Leverkusen, Germany) cages, and were fed a standardized pellet diet taking into account the following: 5-6 grams of food per 100 grams of the rat's body weight per day, keeping in mind a caloric requirement of 60 calories per day. The approximate consumption was 15-20 grams per day. No adjustments to the diet were required to maintain a certain weight; all rats maintained a standard food intake prior to the start of the experimental phase. The diet provided consisted of pellets for laboratory rats (Kruse Feed & Supply, La Habra, California, United States), which meet a standard micro- and macronutrient composition. All the rats received tap water ad libitum.

The rats were divided into two groups with simple randomization, each consisting of 15 rats. For the comparison of the groups, burst pressure and anastomosis dehiscence were analyzed. Anesthetic induction was performed in a chamber containing gauze impregnated with ethyl ether to facilitate subsequent handling.

For the anesthetic process, intraperitoneal anesthesia was applied based on the recommendations for the use of drugs for sedation, anesthesia, and analgesia of the National Institute of Neurology and Neurosurgery "Manuel Velasco Suárez" with ketamine (70 mg/kg/dose) and xylazine (8 mg/kg/dose) until the loss of response reflexes to painful stimuli was observed. After anesthetic induction in an ether chamber, the abdominal cavity was contaminated by intra-abdominal puncture with 3 ml of the dilution solution created with 1 g of fecal material from the same Wistar rat in 10 cc of saline solution.

During this period, the rats were kept in cages at room temperature. A solid diet was excluded. The rats were maintained on a liquid diet with a supply of glucose on free demand. During this period, no type of analgesic and/or antibiotic medication was administered, allowing the progression of the intra-abdominal infection.

**Figure 1:**
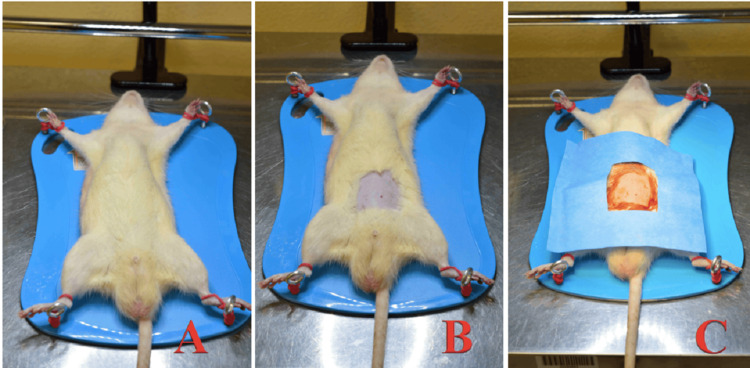
Preoperative preparation. (A) Supine position; (B) Ventral trichotomy; (C) Povidone-iodine asepsis and antisepsis and sterile field placement.

After 18 hours of evolution of the intra-abdominal puncture to cause fecal peritonitis, the first laparotomy was performed (Figure 1). Prior to anesthetic induction in an ether chamber, intraperitoneal anesthesia was applied at the previously mentioned dose until loss of the response to painful stimuli reflexes was visualized. The rats were placed in the supine position, and after trichotomy of the ventral region, asepsis, and antisepsis with povidone-iodine, a sterile field was placed.

**Figure 2:**
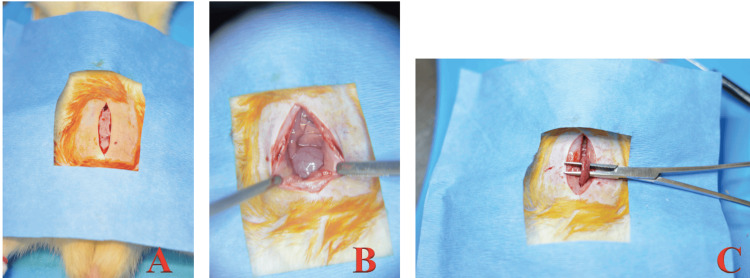
Surgical approach. (A) Celiotomy; (B) Access to the abdominal cavity; (C) Exposure of the colon.

Celiotomy was performed in planes until the entry of the abdominal cavity, washing and drying of the abdominal cavity, location of structures, and exposure of the colon (Figure 2).

**Figure 3:**
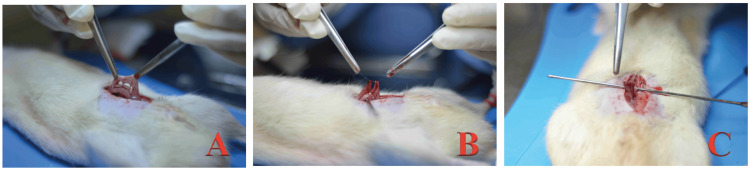
Resection and anastomosis. (A) Exposure of the vascular arcade of the colon; (B) Segment of colon cut, first cardinal point with PDS 6-0; (C) Complete anastomosis.

Subsequently, resection and end-to-end colonic anastomosis were performed with PDS 6-0 (Figure 3) in a plane with four separated simple points.

**Figure 4:**
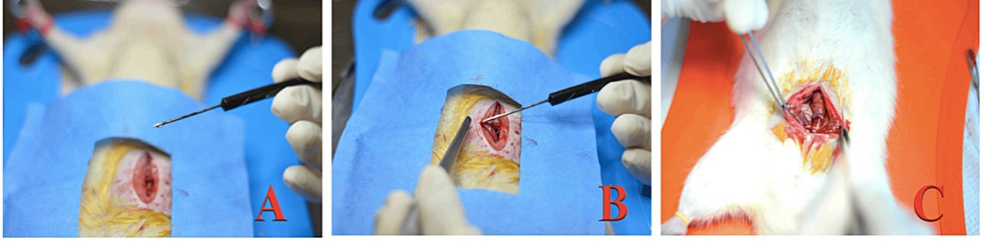
Application of the tissue adhesive. (A) Drop of tissue adhesive; (B) Application of the tissue adhesive over the anastomosis line; (C) Complete anastomosis reinforced with ethyl-2-cyanoacrylate.

The difference in the procedure between the control group and the experimental group was at the end of the anastomosis. At this time, a thin layer of ethyl-2-cyanoacrylate was applied to the anastomosis line, with 30 seconds allowed for the adhesive to dry (Figure 4).

**Figure 5:**
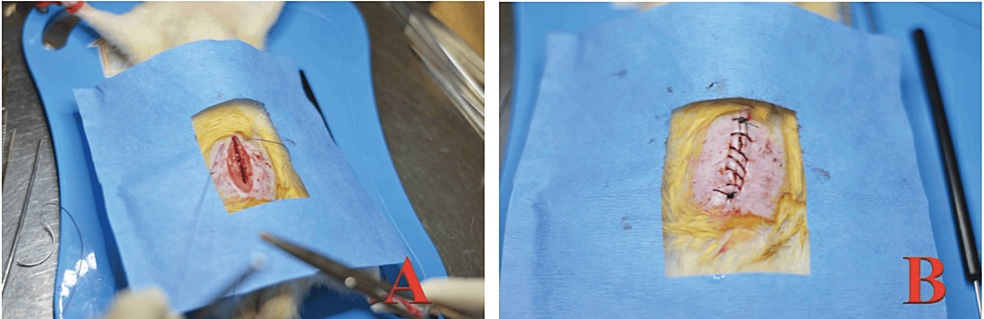
Block wall closure with a Vicryl Plus 3-0 continuous sur-clamp.

We finished the procedure with skin closure and en bloc aponeurosis with Vicryl Plus 3-0 (Ethicon, Inc., Raritan, New Jersey, United States) with a continuous clip (Figure 5).

Postoperative care was given with an adequate room temperature to avoid hypothermia. The rats were maintained on a free water supply with glucose, and the use of a solid diet was excluded. Antibiotics and double analgesics were started when the residual anesthetic effects passed.

**Figure 6:**
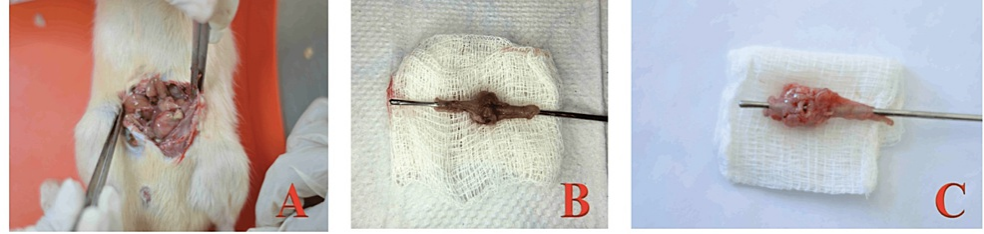
Second laparotomy. (A) Loose and firm adhesions to the anastomoses, fibrin scum, and cloudy, foul-smelling peritoneal fluid; (B) Segment with an intact anastomosis; (C) Segment with a dehiscent anastomosis.

After seven days of the first laparotomy, a second laparotomy was performed to verify the anastomotic integrity visually as the presence or absence of leakage or dehiscence. Subsequently, the anastomosed intestinal segment was resected 2 cm proximal and distal to the anastomosis line for a burst pressure test (Figure 6). After resection, the rat was sacrificed.

**Figure 7:**
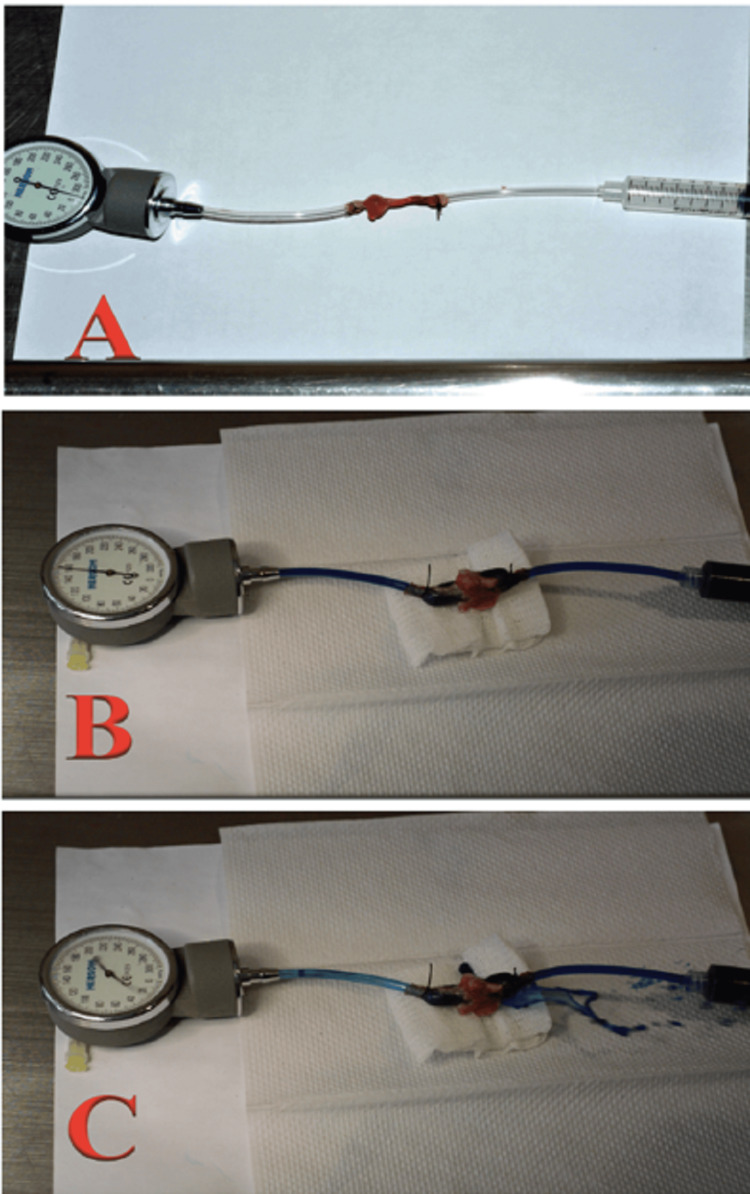
Burst test. (A) Segment of colonic anastomosis connected to a manometer and syringe; (B) Complete anastomosis with passage of methylene blue. Note the distension capacity of the anastomosis, in addition to the pressure marked on the manometer (160 mmHg); (C) Timing of loss of strength of the anastomosis. Here, a methylene blue leak is observed accompanied by an abrupt drop in the pressure marked on the manometer.

For the burst pressure test, resection of the colonic anastomosis segment was performed, considering 2 cm before and 2 cm after the anastomosis. A catheter for infusion of methylene blue solution was inserted into the proximal end of the colon segment, and a catheter connected to a manometer was inserted into the distal end. Both intestinal ends were ligated with 3-0 silk to ensure a watertight compartment. Subsequently, a diluted methylene blue solution was infused through the catheter via a 20 ml syringe. A sign of anastomosis leakage was the presence of a blue-stained fluid discharge accompanied by a sudden drop in pressure marked by the manometer. The burst pressure of the anastomosis was defined as the pressure at the time of observing the methylene blue leak (Figure 7).

Statistical analysis

For statistical analysis, GraphPad Prism version 9.3.1.471 (Dotmatics, Boston, Massachusetts) was used. For the descriptive statistics of the qualitative variables, both the absolute frequencies and the relative frequencies were calculated, and the proportions of the dichotomous categorical variables were placed in a 2x2 contingency table for comparison using the chi-square test. For quantitative variables, measures of central tendency and dispersion (mean, median, and standard deviation) were used.

Regarding the inferential statistics of the quantitative variables, first, their normality in the Gaussian distribution was assessed using the Anderson‒Darling and Shapiro‒Wilk tests. Once the results were obtained, the comparison between unpaired groups was carried out with Student's t test for parametric data and the Mann‒Whitney U test for nonparametric data. In all analyses, p-values less than 0.05 with a 95% confidence interval (CI) were considered significant.

## Results

We included 30 rats in total for the entire study, which were randomized into groups of 15 rats each, for a total of two groups (control and experimental). Both groups were exposed to fecal-induced peritonitis for 18 hours (see Materials and Methods section); no events occurred that modified or affected the surgical procedure in any of the subjects in each group. During the time of the study, no deaths occurred, and all subjects were able to complete the study procedures.

All the rats were male, with a median age of five months (range: four to six months). The mean weight for the experimental group was 350.10 g (SD ±28.20) compared to the control group, which was 350.80 g (SD ±25.26); without statistically significant differences between both groups (p=0.9407). Regarding the parameters of interest evaluated, two rats (13.33%) presented dehiscence of the anastomosis in the experimental group vs eight (53.33%) in the control group, with a statistically significant difference (p=0.0201). Of the rats that did not present dehiscence in both groups (13 in the experimental group vs. seven in the control group), burst pressure was evaluated at 102.3333 mmHg (SD ±75.19) for the experimental group vs 45.0667 mmHg (SD ±69.44) for the control group. Although there is an apparent difference between both groups, it was not statistically significant (p=0.5350). Table 1 shows the results of the comparisons between groups in terms of weight, burst pressure, and dehiscence of the anastomosis.

**Table 1:** Results of the comparison between groups in relation to weight, dehiscence, and burst pressure. SD: standard deviation; CA: cyanoacrylate

		Experimental group, Ethyl-2-CA (n=15)		Control group (n=15)		p-value
		Mean	SD ±	Mean	SD ±	
Weight (g)		350.10	28.20	350.80	25.26	0.9407
		Mean, n=13	SD ±	Mean, n=7	SD ±	
Burst pressure (mmHg)		102.3333	75.19	45.0667	69.44	0.5350
		Number	%	Number	%	
Anastomosis dehiscence	Yes	2	13.33	8	53.33	0.0201
	No	13	86.67	7	46.67	

**Figure 8:**
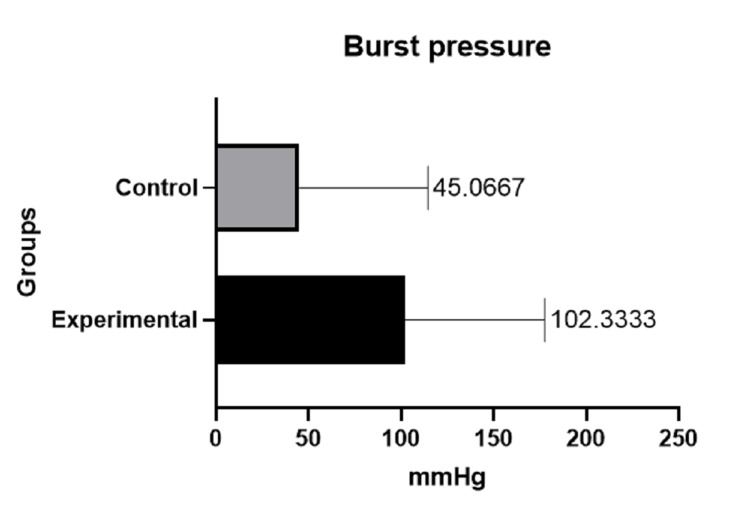
Difference in mean burst pressure between the control group and the experimental group.

**Figure 9:**
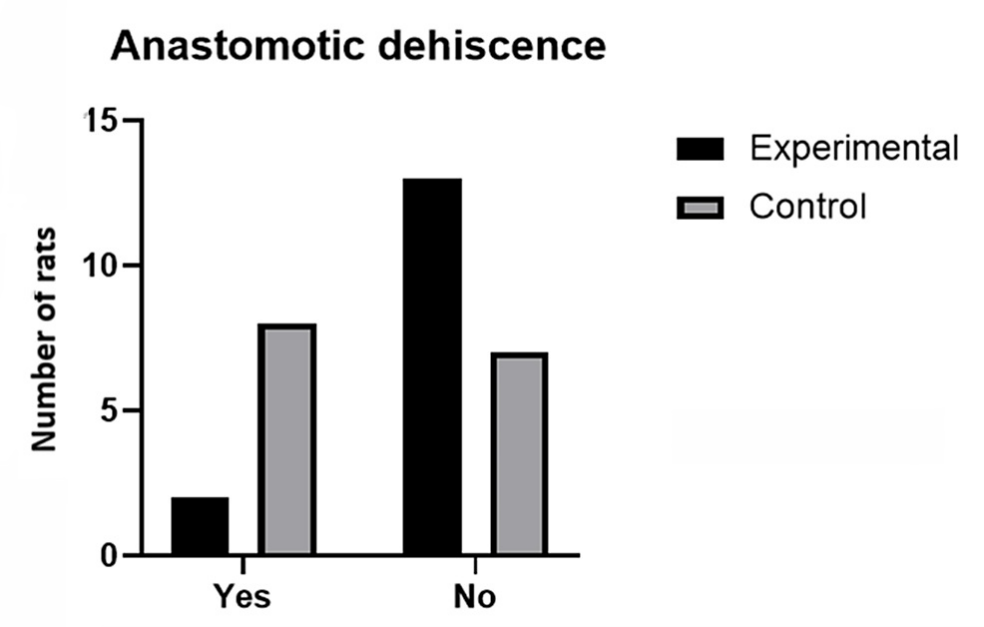
Proportion of dehiscence presented in the control group and in the experimental group.

Figure 8 and Figure 9 show, graphically, the differences in both parameters evaluated (burst pressure and anastomosis dehiscence, correspondingly).

## Discussion

As seen in the results, when comparing the dehiscence between the groups, a 50% decrease in dehiscence is observed in the group treated with Ethyl-2-CA, which contrasts with what has been reported in the literature [19].

The direct use of CAs on abdominal organs has been studied in several trials, including one trial comparing the repair of traumatic liver injuries in Wistar rats against a fibrin sealant [19]. The results showed that although the inflammatory reaction was similar in both groups, the fibrin-treated group showed signs of greater neovascularization and a greater presence of type I and III collagen fibers, indicating better tissue repair. CAs have also been successfully applied to traumatic wounds of the spleen or small intestine. The use of N-butyl-2-CA in the primary repair of penetrating wounds in the abdominal viscera is an alternative to standard treatments [19].

In contrast, when using CAs, specifically, N-butyl-2-CA, in colon anastomoses in rats, as mentioned in the review by García Cerda et al., some studies found a higher number of adhesions in the groups treated with adhesives, as well as a stronger inflammatory reaction and less resistance to anastomosis than those observed in the suture groups [19]. CAs did not improve the healing process and worsened recovery in the first week after the intervention [19]

For our study, an 18-hour evolution of fecal peritonitis was considered for the evaluation of dehiscence, which is relevant due to the evidence shown in the proof of concept study carried out by Espinoza-Anguiano, where there was an increase in the incidence of anastomotic dehiscence according to the time interval the animal model was exposed to an intra-abdominal infectious process [14]. This study highlights a 66% incidence of dehiscence after 16 hours of evolution of fecal peritonitis [14].

Souza and Oliveira et al. concluded that ethyl-CA was better tolerated in rat skin closure without inducing necrosis, allergic reactions, or infections, presenting several advantages over octyl-CA [28].

The use of octyl-CA has also been evaluated for normal and high-risk colonic anastomoses in rats [19]. After evaluating the degree of inflammation and healing, it was concluded that in the experimental model used, the application of the adhesive did not produce any additional benefit compared to conventional suture and caused a more severe inflammatory reaction, which hindered healing [19].

Elemen et al. carried out an experimental study in 96 male Sprague‒Dawley rats divided into two groups to compare the use of different materials for anastomosis: one with polyglactin 910 and the other with ethyl-2-CA [15]. In their results, no significant differences were observed between burst pressures between each subgroup on postoperative days 2 and 6. The authors concluded that better healing was observed with the use of ethyl-2-CA, with less time to perform the anastomosis and the same tensile strength.

Another study compared the results of rat intestinal anastomoses using CAs against polyglactin 910 [24]. After evaluating the tensile strength, degree of healing, and other aspects of toxicity/healing, the authors emphasized that the duration of surgery for the anastomoses was shorter, the procedure was cleaner with CA, and no significant differences in the burst pressure between the two materials were found [24]. In our study, we did not observe a significant difference between the groups in relation to burst pressure.

The main disadvantages when using a CA are a consequence of the lack of studies in certain specific situations [18], such as (1) in areas of high tension, where it is advisable to use some deep approximation sutures to reduce tension and facilitate contact of the surfaces to be joined; (2) on incisions larger than 5 cm, in which its effect has not yet been documented; (3) in contaminated or infected areas; (4) in patients with abnormal healing; and (5) inside the mucosa [18].

Regarding the limitations found in our study, it is important to mention that the TA applicator is not adapted for such a small tissue, making the TA application a complex process and posing the risk of exceeding its recommended quantity or causing its spillage into adjacent tissues. Another limitation is that, since it is an experimental model in rodents, the results are not entirely transferable to what could happen in a clinical context; however, animal models serve as an important starting point for the planning of future human research that can confirm these results.

## Conclusions

The use of ethyl-2-CA as reinforcement in colonic anastomoses in the presence of fecal peritonitis decreases the frequency of anastomosis dehiscence in our murine model, presenting a burst pressure similar to that reported using sutures alone. These results are encouraging to continue experimentation and search for new tools to improve surgical outcomes associated with suture materials.

In general, experimentation in search of new and better suture materials is very little explored and we believe that it is of great importance to increase the available options, which must be in accordance with the surgical needs presented by the specific clinical case.
